# The Novel-m0230-3p miRNA Modulates the CSF1/CSF1R/Ras Pathway to Regulate the Cell Tight Junctions and Blood–Testis Barrier in Yak

**DOI:** 10.3390/cells13151304

**Published:** 2024-08-05

**Authors:** Qiu Yan, Qi Wang, Yong Zhang, Ligang Yuan, Junjie Hu, Xingxu Zhao

**Affiliations:** 1 College of Veterinary Medicine, Gansu Agriculture University, Lanzhou 730070, China; zhangyong@gsau.edu.cn (Y.Z.); yuan2918@126.com (L.Y.); hujj@gsau.edu.cn (J.H.); zhaoxx@gsau.edu.cn (X.Z.); 2 Gansu Key Laboratory of Animal Generational Physiology and Reproductive Regulation, Lanzhou 730070, China

**Keywords:** yak, novel-m0230-3p, tight junctions, BTB, CSF1/CSF1R/Ras signaling pathway

## Abstract

The yak (Bos grunniens) is a valuable livestock animal endemic to the Qinghai–Tibet Plateau in China with low reproductive rates. Cryptorchidism is one of the primary causes of infertility in male yaks. Compared with normal testes, the tight junctions (TJs) of Sertoli cells (SCs) and the integrity of the blood–testis barrier (BTB) in cryptorchidism are both disrupted. MicroRNAs are hairpin-derived RNAs of about 19–25 nucleotides in length and are involved in a variety of biological processes. Numerous studies have shown the involvement of microRNAs in the reproductive physiology of yak. In this study, we executed RNA sequencing (RNA-seq) to describe the expression profiles of mRNAs and microRNAs in yaks with normal testes and cryptorchidism to identify differentially expressed genes. GO and KEGG analyses were used to identify the biological processes and signaling pathways which the target genes of the differentially expressed microRNAs primarily engaged. It was found that novel-m0230-3p is an important miRNA that significantly differentiates between cryptorchidism and normal testes, and it is down-regulated in cryptorchidism with *p* < 0.05. Novel-m0230-3p and its target gene CSF1 both significantly contribute to the regulation of cell adhesion and tight junctions. The binding sites of novel-m0230-3p with CSF1 were validated by a dual luciferase reporter system. Then, mimics and inhibitors of novel-m0230-3p were transfected in vitro into SCs, respectively. A further analysis using qRT-PCR, immunofluorescence (IF), and Western blotting confirmed that the expression of cell adhesion and tight-junction-related proteins Occludin and ZO-1 both showed changes. Specifically, both the mRNA and protein expression levels of Occludin and ZO-1 in SCs decreased after transfection with the novel-m0230-3p mimics, while they increased after transfection with the inhibitors, with *p* < 0.05. These were achieved via the CSF1/CSF1R/Ras signaling pathway. In summary, our findings indicate a negative miRNA-mRNA regulatory network involving the CSF1/CSF1R/Ras signaling pathway in yak SCs. These results provide new insights into the molecular mechanisms of CSF1 and suggest that novel-m0230-3p and its target protein CSF1 could be used as potential therapeutic targets for yak cryptorchidism.

## 1. Introduction

Yak is a unique bovine species endemic to the high altitudes of Central Asia and of significant economic, cultural, and scientific value [1,2]. Cryptorchidism, a common disease of the male reproductive system, is one of the chief causes of infertility in yaks [3]. The current understanding about the causes of cryptorchidism is that the location of the cryptorchidism in the abdominal cavity and subcutaneous fat layer, and that changes in the environment of the stromal cells, destroy the tight connection between Sertoli cells (SCs), adversely affecting intercellular adhesion. Ultimately, this leads to the destruction of the blood–testis barrier (BTB) [4]. The mechanism underlying the molecular regulation of cryptorchidism is poorly understood. Spermatogenesis is a multicellular process that is precisely coordinated by the main somatic cells of the testis, namely, the SCs and Leydig cells (LCs) [5]. This process is dependent on the proliferation, tight junctions, and adhesion functions of SCs, with adhesion between SCs and adjacent cells providing a bridge for the exchange of both materials and information between cells, as well as an immune barrier for spermatogenesis [6]. 

The testis is an organ with strong transcriptional activity, and its development is regulated by many non-coding RNAs (ncRNAs) [7]. MicroRNAs (miRNAs) are a category of endogenous single-stranded non-coding ncRNAs of approximately 19–25 nucleotides in length, which can regulate the post-transcription expression of genes. Numerous studies have shown that miRNAs are involved in the regulation of cell proliferation, differentiation, senescence, apoptosis, and autophagy, as well as other biological processes such as reproduction, immune adhesion, and TJs [8,9]. There is growing evidence that miRNAs can regulate the proliferation and adhesion functions of SCs by the targeting and regulation of specific genes [10,11,12,13]. Several miRNAs have been found to be either specifically or highly expressed in SCs [14]. However, there have been few studies on the miRNAs involved in reproductive immunity, with most of these focusing on the female reproductive tract rather than the testis [15,16,17], and there is minimal information on the regulation of yak cryptorchidism. 

RNA sequencing (RNA-seq) has emerged as a powerful tool for the identification and characterization of genes, and it can be used for the in-depth screening of mRNA-miRNAs connected with diseases of the reproductive system and for understanding the complex pathogenesis of these diseases [18,19]. However, relatively little is known of miRNAs and their functions in large livestock species, particularly the yak. Hence, it is fundamental to inquire the miRNA and mRNA expression profiles in yak testes to understand their functions and regulatory mechanisms, as well as to identify the key targets of these miRNAs in the processes underlying testicular development, spermatogenesis, cryptorchidism, and cell TJs.

In the present research, an RNA-seq analysis and a series of molecular biology experiments were the capable tools used to ascertain differentially expressed (DE) genes in both the normal testes and cryptorchidism of yak. A novel miRNA, namely novel-m0230-3p, was identified, which was shown to target CSF1 to modulate cell adhesion and TJs. Macrophage colony-stimulating factor (CSF1) is a considerable growth factor required for controlling cell survival, differentiation, proliferation, and renewal [20]. In the reproductive system, CSF1 stimulates SCs to produce glial cell-derived neurotrophic factor (GDNF), and it stimulates stem cell proliferation and adhesion [21]. We attempt to investigate whether novel-m0230-3p may further influence a wide range of male reproductive events in the yak, such as spermatogenesis, infertility, and the occurrence of cryptorchidism, by modulating the expression and specific molecular mechanisms of cell adhesion and TJ-related proteins in SCs. 

## 2. Materials and Methods

All animal protocols were conducted in accordance with the Animal Care and Use Committee of the School of Veterinary Medicine of the Gansu Agricultural University (approval number: GSAU-Eth-VMC-2023-036).

### 2.1. Experimental Animals and Samples

Six healthy male Tianzhu white yaks, with an average age of 3–4 years, from a local slaughterhouse in Tianzhu County, Gansu Province, China, were used in this study. The yaks were divided into two groups: the normal testis group (Tes; *n* = 3) and the cryptorchidism group (Cry; *n* = 3). All samples (normal or cryptorchidism testicular tissue) were harvested immediately after the sacrifice of the animals and immediately washed with sterile phosphate-buffered saline (PBS) containing 100 U/mL penicillin and 100 mg/mL streptomycin. The animals were shocked and became unconscious before being sacrificed and then slaughtered according to standard procedures at that slaughterhouse. The tissue was then decapsulated and separated into small pieces and snap-frozen in liquid nitrogen before being stored at −80 °C for subsequent RNA and protein extraction or fixed in 4% neutral paraformaldehyde for histological analysis.

### 2.2. Library Preparation

The detailed methods for obtaining the strand-specific cDNA library for miRNA-seq and mRNA-seq were previously described [22]. The cDNA libraries from the two groups were sequenced on the Illumina HiSeqTM 250 and HiSeqTM 4000 platforms by Gene Denovo Biotechnology Co., Ltd. (Guangzhou, China).

### 2.3. RNA Extraction and Identification of miRNAs and mRNAs in Normal Testis and Cryptorchidism of Yak by RNA-Seq

According to the manufacturer’s instructions, total RNA was extracted from each testis sample using the Trlquick Reagent (R1100, Solarbio, Beijing, China). The quality and concentration of the RNA were evaluated on a NanoDrop spectrophotometer (Thermo Fisher, Waltham, MA, USA), an Agilent 2100 Bioanalyzer (Agilent Technologies, Santa Clara, CA, USA), and RNase-free agarose gel electrophoresis. All samples had an RNA integrity number (RIN) > 7.5. A TruSeq Small RNA Sample Prep Kit and Ribo-ZeroTM rRNA Removal Kit (Illumina, San Diego, CA, USA) were used to synthesize miRNA and mRNA libraries. Fragments per kilobase of exon per million mapped fragments (FPKM) were calculated to determine the miRNA and mRNA expression levels using StringTie v2.2.0 [23]. mRNA expression levels were quantified by using the FPKM method [24]. The miRNA expression level was calculated and normalized to TPM (TPM = actual miRNA count/total count of clean reads × 10^6^). DE mRNAs and miRNAs were identified using the edgeR package (version 4.4) (http://www.bioconductor.org/packages/release/bioc/html/edgeR.html (accessed on 11 April 2024)). Differential expression of miRNAs and mRNAs was defined using the criteria of |log2 fold-change (FC)| > 1 and *p* < 0.05 [25].

### 2.4. GO and KEGG Pathway Analysis 

Gene Ontology (GO) annotation and Kyoto Encyclopedia of Genes and Genomes (KEGG) enrichment analysis were performed for all DE mRNAs and miRNAs using the GO database (http://www.geneontology.org/ (accessed on 11 April 2024)) and KEGG database (http://www.genome.jp/kegg/pathway.html (accessed on 11 April 2024)), respectively.

### 2.5. Dual-Luciferase Reporter Assays

The wild-type (WT) CSF1 3′-UTR fragments containing the putative novel-m0230-3p binding site and its corresponding mutant type (Mut) were designed and synthesized by Zebra Biotechnology (Changsha, China) and cloned into the pmirGLO cloning vector between the *Mlu I* and *Xho I* cloning sites. The novel-m0230-3p mimic, mimic NC, inhibitor-m0230-3p, and inhibitor NC were all purchased from Sangon Biotech (Shanghai, China), and their sequences are provided in . The luciferase reporter plasmids were transfected with the novel-m0230-3p mimic or mimic NC into 293T cells using Lipofectamine 2000 (Thermo Fisher, Waltham, MA, USA). After transfection for 48 h, the luciferase activity was detected using a Dual-Luciferase Reporter Assay Kit (Promega, Madison, WI, USA). 

### 2.6. Hematoxylin–Eosin Staining 

The tissue was fixed, washed, dehydrated, and paraffin-embedded according to a routine histopathology protocol [26]. H&E staining was performed using a Hematoxylin-Eosin/HE Staining Kit (G1120, Solarbio) according to the specific experimental methods described in great detail in our previous paper [3]. Finally, the slides were examined under light microscopy and imaged using a digital camera (BX51TRF, Olympus, Tokyo, Japan).

### 2.7. Cell Culture and Transfection

Sertoli cells were isolated as previously described with minor modifications [27]. Briefly, the testis tissue was minced into a paste and digested with 1% type IV collagenase mixed with 0.25% trypsin for about 0.5 h at 37 °C, after which digestion was terminated with high-glucose DMEM containing 10% FBS. The digested cells were filtered through a 200-mesh screen. After centrifugation and washing, the SCs were cultured in high-glucose DMEM supplemented with 10% fetal bovine serum (FBS) containing streptomycin (50 μg/mL) and penicillin (50 IU/mL) at 37℃ in a 5% CO_2_ atmosphere. The medium was removed and replaced with fresh medium after about 48 h. 

### 2.8. Quantitative Reverse-Transcription PCR 

RNase-free consumables were prepared, and the total RNA was extracted from the tissue and cells. First-strand cDNA for mRNA or miRNA was synthesized from 500 ng of each total RNA sample using an Evo M-MLV RT Kit with gDNA Clean or miRNA 1st strand cDNA synthesis kit (Accurate Biology, Changsha, China) according to the instructions of the respective kits. qRT-PCR was conducted using 2 × SYBR Green qPCR Master Mix (B21202, Bimake, Houston, TX, USA) on the LightCycler 96 Real-time System (Roche, Basel, Switzerland). The two-step amplification program was as follows: 95 °C for 600 s and 40 two-step amplification cycles of 95 °C for 15 s and 60 °C for 45 s. The expression of the housekeeping genes GAPDH or U6 were measured for normalization. Table S1 lists the sequences of the specific primers, which were designed and synthesized by AZENTA Life Sciences (Tianjin, China) and Tsingke Biotechnology (Beijing, China). These experiments were performed in triplicate, and relative expression was calculated using the 2^−ΔΔCt^ method [28].

### 2.9. Immunofluorescence

The paraffin sections (4 μm) were cut using the semi-automatic rotary microtome (HistoCore MULTICUT, LEICA, Heidelberg, Germany) and treated as previously described [29]. The sections were deparaffinized with conventional gradient alcohol and washed with PBS. Then, the sections were antigen-repaired by using citrate buffer. After blocking with 5% BSA at room temperature for 45 min, the primary and fluorescent secondary antibodies were incubated according to the antibody conditions. After washing with PBS, the sections were blocked with Antifade Mounting Medium (P0126, beyotime, Shanghai, China). 

Cells were fixed in 4% paraformaldehyde for at least 2 h and then washed with 4 °C ice-cold PBS. Then, after 30 min of permeabilization with 0.5% Triton X-100, the cells were washed with ice-cold PBS and blocked with 5% bovine serum albumin (A8010, Solarbio) for 30 min at room temperature, with both permeabilization and incubation being carried out at room temperature. The cells were incubated overnight at 4 °C with rabbit anti-MCSF (1:80, T55362, Abmart, Shanghai, China), rabbit anti-WT1 (1:100, 12609-1-AP, Proteintech, Wuhan, China), rabbit anti-SOX9 (1:100, bs-10725R, Bioss, Beijing, China), or rabbit anti-CSF1R (1:100, TP70252, Abmart), while the sections were incubated overnight at 4 °C with rabbit anti-β-catenin (1:100, 51067-2-AP, Proteintech) or rabbit anti-ZO1 (1:100, 21773-1-AP, Proteintech). This was followed by incubation with the secondary goat anti-rabbit IgG H&L (Alexa Fluor^®^647) antibody (1:200, ab150079, Abcam, Cambridge, UK) or goat anti-mouse IgG H&L (Alexa Fluor^®^ 488) secondary antibody (1:200, ab150113, Abcam) at 37 °C for 1 h in the dark. Cell nuclei were stained with 1 μg/mL 4′,6-Diamidino-2-phenylindole (DAPI) (D9542, Sigma-Aldrich, St. Louis, MO, USA). Digital images were acquired using Echo Revolve anteroposterior/inverted microscope integrated imaging system (Revolve Omega, M-00163-RVL, California Echo, CA, USA).

### 2.10. Western Blotting

The total protein was extracted from tissues and cells using RIPA buffer (R0010, Solarbio) containing 1% PMSF (P0100, Solarbio). Western blotting was performed as previously described [29]. Briefly, 40 μg of protein from each sample was separated with 12% SDS-PAGE gels, and the bands were electro-transferred onto PVDF membranes (IPVH00010, Millipore, Billerica, MA, USA). After blocking, the membranes were incubated overnight at 4 °C with rabbit anti-MCSF (1:1000), rabbit anti-βcatenin (1:2000), rabbit anti-CSF1R (1:1000), rabbit anti-Ras (1:1000, ab52939, Abcam), rabbit anti-ZO1(1:5000), rabbit anti-Occludin (1:4000, T55997, Abmart), and mouse anti-GAPDH (1:5000, M20006, Abmart). After washing with PBST, the membranes were incubated with the secondary antibodies, horseradish peroxidase (HRP)-conjugated goat anti-rabbit (1:5000, bs-0295G-HRP, Bioss), or goat anti-mouse secondary antibody (1:5000, bs-0368G-HRP, Bioss) at 37 °C for 1 h. Protein bands were visualized with enhanced chemiluminescence solution (Abnova, Taipei, Taiwan, China), and signals were quantified using ImageJ 1.48v (NIH, Bethesda, Maryland, MD, USA).

### 2.11. Statistical Analysis

Statistical analysis was performed with SPSS version 22.0 (IBMS Corp., Armonk, NY, USA). The data are expressed as the mean ± SD and are visualized using GraphPad Prism version 5.0 (La Jolla, CA, USA). Data were analyzed using *t*-tests (two groups) or one-way ANOVA (multiple groups). The differences between the two groups are annotated as *p* < 0.05 and *p* < 0.01.

## 3. Results

### 3.1. Cryptorchidism Is Associated with Disruption of BTB and Abnormal Expression of TJ Proteins

**Figure 1:**
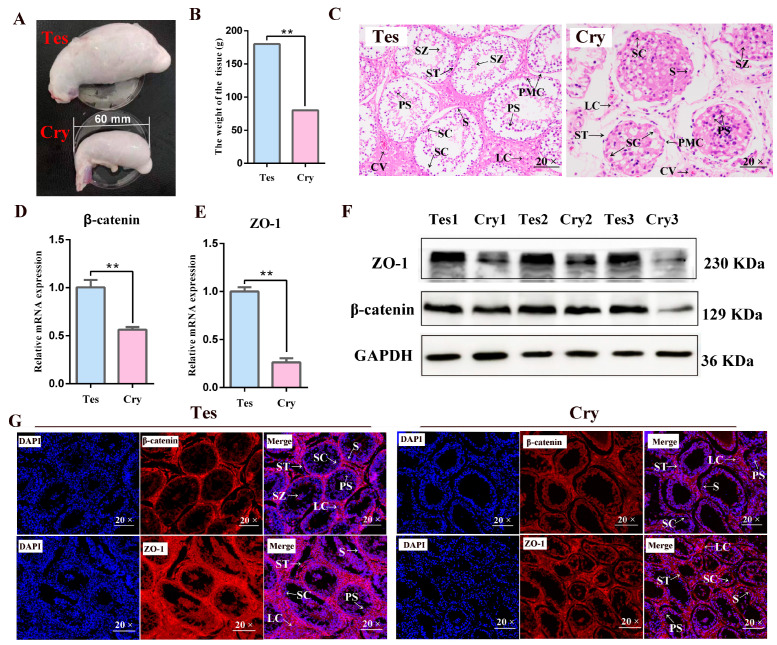
Comparison of appearance and pathology of normal testis and cryptorchidism in yak. (**A**,**B**) Testes weights, ** *p* < 0.01. (**C**) H&E staining, magnification of 20×. (**D**) mRNA expression of β-catenin in normal testis and cryptorchidism of yak. Values represent mean ± SD; *n* = 3, ** *p* < 0.01. (**E**) mRNA expression of ZO-1 in normal testis and cryptorchidism of yak. Values represent mean ± SD; *n* = 3, ** *p* < 0.01. (**F**) Protein expression of β-catenin and ZO-1 in normal and cryptorchid testes of yaks. (**G**) Localization of β-catenin and ZO-1 proteins in yak testes, analyzed by immunofluorescence staining. β-catenin and ZO-1 in tissue are shown separately in red and nuclei are colored blue; magnification, 20×. Tes: normal testis; Cry: cryptorchidism; S: spermatogonium; SZ: spermatozoa; ST: seminiferous tubule; PS: primary spermatocyte; SC: Sertoli cell; LC: Leydig cell, PMC: peritubular myoid cell; CV: capillary vessel.

The weights of one unilateral normal testis and one unilateral cryptorchidism were determined separately. The weight of the cryptorchidism was 80 g, which was significantly reduced compared to that of the normal testis, which was 180g (*p* < 0.01) (Figure 1A,B). The H&E-stained tissue sections showed that the lumen of seminiferous tubules (STs) in the normal testis was significantly larger than that in cryptorchidism, with greater numbers of both SCs and LCs, and the LCs are large with abundant cytoplasm and prominent nuclei. The STs of the normal testis were intact, and the lumen was smooth; however, in cryptorchidism, the lumen of the STs was severely atrophied. Furthermore, peritubular myoid cells were present around the lamina propria of the STs, and spermatogonial cells were arranged in multiple layers in the normal testis. In the lamina propria of cryptorchidism, the number of spermatophores was reduced, and some of them were shed in the lumen (Figure 1C). Furthermore, we examined the expression of TJ-related proteins in both groups, using qRT-PCR to measure the mRNA while the protein expression was measured by Western blotting and immunofluorescence. It was found that the mRNA and protein levels of ZO-1, also known as tight junction protein 1 (TJP1) in yak, and β-catenin were both significantly reduced in cryptorchidism (*p* < 0.01) (Figure 1D–F). The cellular expression of these proteins was then examined using immunofluorescence staining, showing that β-catenin protein was mainly distributed in the SCs, LCs, and myoid cells, with minimal expression in germ cells, while the ZO-1 protein was mainly expressed in the LCs with small amounts in the SCs (Figure 1G). 

### 3.2. Overview of mRNA Sequencing Datasets and Functional Analysis in Normal Testis and Cryptorchidism of Yak

**Figure 2:**
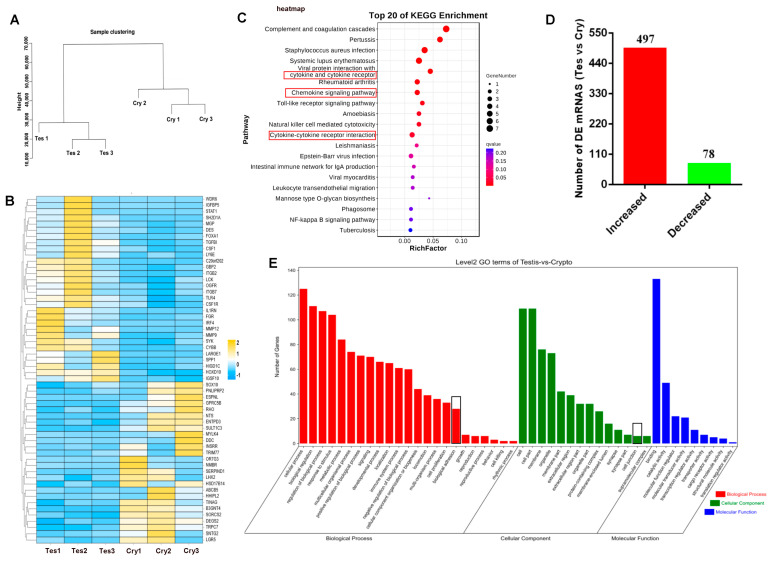
Functional annotation and enrichment analyses in normal testis and cryptorchidism. (**A**) Cluster analysis of six samples. (**B**) Clustering heatmap of differential mRNA expression. (**C**) KEGG pathway analysis of source genes of differential mRNAs. (**D**) Number of DE mRNAs, with red indicating upregulation and green indicating downregulation. (**E**) GO enrichment analysis of DE mRNAs. Tes: normal testis, Cry: cryptorchidism.

**Figure 3:**
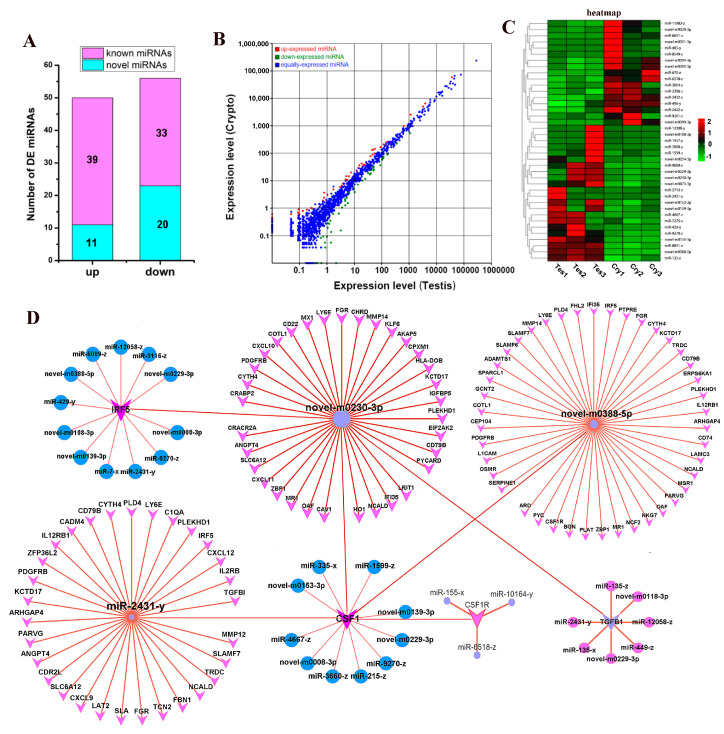
miRNA–mRNA interaction network. (**A**) Number of DE miRNAs, with purple indicating known and blue indicating novel. (**B**) Expression levels of DE miRNAs. (**C**) Clustering heatmap of DE miRNA expression. Red corresponds to upregulation, and green corresponds to downregulation. (**D**) Network plot of novel-m0230-3p and its target gene *CSF1*. Tes: normal testis, Cry: cryptorchidism.

Averages of 37,546,760 and 38,773,819 raw reads were obtained for the normal testis and cryptorchidism libraries, respectively. After filtering, we finally obtained 35,082,700 (93.43%) and 35,929,045 (92.66%) small RNA clean tags, respectively, for subsequent analysis (Figure 2A). mRNA and miRNA libraries were constructed for both groups and sequenced using RNA-seq to investigate the expressions of mRNAs and miRNAs. Using the criteria of *p* < 0.05 and FC > 1.5, a comparison of the normal testis and cryptorchidism showed that 575 genes were differentially expressed, of which 497 were upregulated and 78 were downregulated in the cryptorchidism samples (Figure 2D) (Table S2). A heatmap of the DE mRNAs showed excellent repeatability for both groups (Figure 2B). GO and KEGG enrichment analyses showed that most DE genes were mainly involved in biological processes and pathways related to cytokine–cytokine receptor interaction, cell growth, reproduction, immunity, metabolism, adhesion, and cell TJs (Figure 2C–E). Similarly, a comparison of the two groups showed the differential expression of 103 miRNAs, of which 50 were upregulated (39 known miRNAs and 11 novel miRNAs) and 53 were downregulated (33 known miRNAs and 20 novel miRNAs) in the cryptorchidism samples (Figure 3A,B) (Table S3). The heatmap of the DE miRNAs showed excellent repeatability for the normal testis and cryptorchidism groups (Figure 3C). 

### 3.3. Screening and Functional Analysis of miRNA-mRNA in Normal Testis and Cryptorchidism of Yak

To identify the potential miRNA target transcripts involved in the homeostatic regulation of testicular immune adhesion and cell TJs, including the BTB, the expression profiles of DE miRNAs and the BTB-related DE mRNAs were combined and analyzed. The key potential regulatory networks of the miRNA-targeted genes associated with the testis BTB were visualized using the Cytoscape software (version 3.7.1). Four genes associated with testicular adhesion and cell TJs [CSF1, CSF1R, transforming growth factor beta 1 (TGFB1), and interferon regulatory factor 5 (IRF5)] were predicted to be regulated by all 13 miRNAs, with CSF1R, TGFB1, and IRF5 showing co-regulation by 3, 8, and 13 miRNAs, respectively. Notably, CSF1, CSF1R, TGFB1, and IRF5 have been demonstrated to be associated with cell adhesion or tight junctions based on our literature review [30,31,32,33,34]. miR-2431-y, novel-m0230-3p, and novel-m0388-5p, which were predicted to be associated with cell immune adhesion and TJs, were predicted to regulate the expression of 31, 36, and 44 DE mRNAs, respectively (Figure 3D) (Table S4).

### 3.4. Analysis of DE Genes and miRNAs in Normal Testis and Cryptorchidism of Yak Using qRT-PCR and RNA-Seq

**Figure 4:**
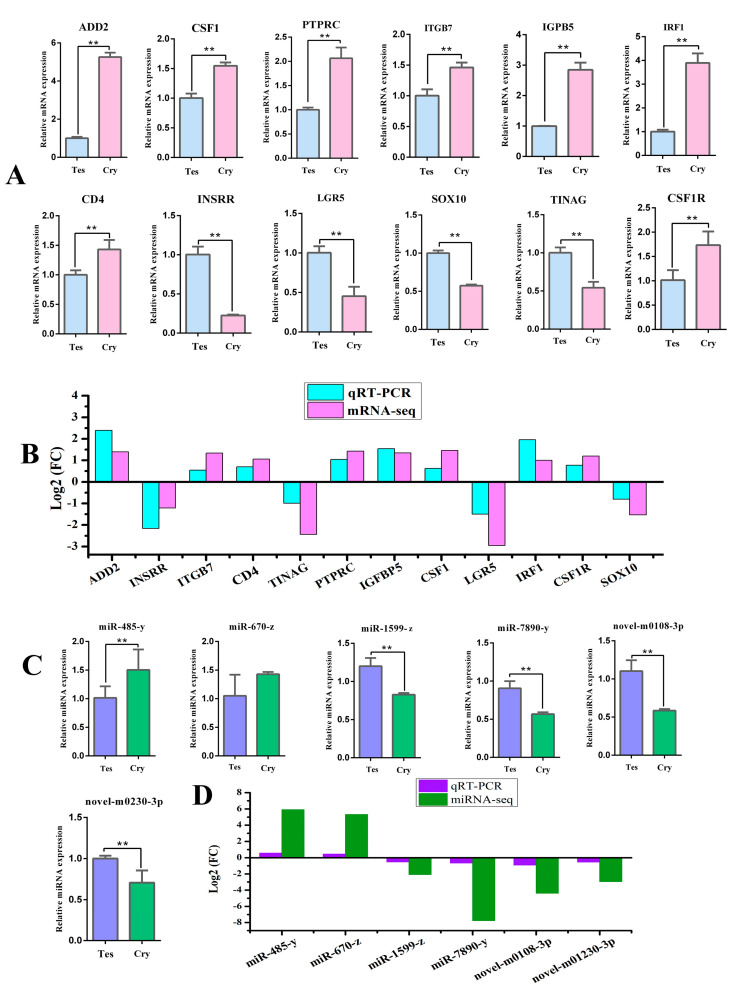
qRT-PCR verification of DE genes in normal testis and cryptorchidism in yak. (**A**) mRNA expression of DE genes shown by qRT-PCR analysis. (**B**) Comparison of |log2 FC| expression levels of mRNA-seq and qRT-PCR. (**C**) mRNA expression of DE miRNAs shown by qRT-PCR analysis. (**D**) Comparison of |log2 FC| expression levels of miRNA-seq and qRT-PCR. Values represent mean ± SD; *n* = 3. ** *p* < 0.01. FC, fold-change. Tes: normal testis, Cry: cryptorchidism.

Twelve DE mRNAs and six DE miRNAs were randomly selected to verify the accuracy of the mRNA-seq and miRNA-seq results by a qRT-PCR analysis. The expression trends in mRNA and miRNA followed those of RNA-seq in terms of significant differences (*p* < 0.05 or *p* < 0.01), indicating that the RNA-seq results were reliable for identifying DE mRNAs and miRNAs (Figure 4A–D).

### 3.5. Verification of CSF1 and CSF1R Protein Levels by Western Blotting and Immunofluorescence 

**Figure 5:**
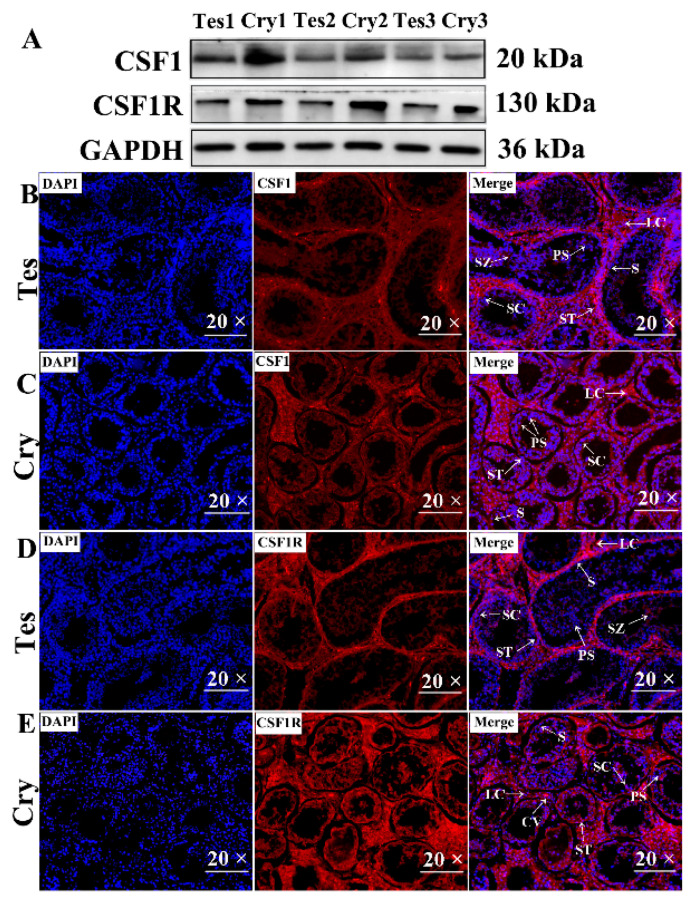
Verification of CSF1 and CSF1R. (**A**) Expression patterns of CSF1 and CSF1R proteins by Western blot analysis; *n* = 3. (**B**–**E**) Immunofluorescence assay for expression and location of CSF1 and CSF1R in testis and cryptorchidism; magnification, 20×. Tes: normal testis; Cry: cryptorchidism; S: spermatogonium; SZ: spermatozoa; ST: seminiferous tubule; PS: primary spermatocyte; SC: Sertoli cell; LC: Leydig cell, CV: capillary vessel.

To confirm the abundance of variation in the protein levels, we chose the potential target protein CSF1 and its receptor CSF1R for Western blotting and IF assays. The target proteins were differentially expressed in the testis and cryptorchidism, and the expression tendency and expression levels were similar to that of the mRNA of CSF1 and CSF1R, which were validated in Section 3.4 (Figure 4A). The CSF1 and CSF1R protein levels were significantly upregulated (Figure 5A). We further verified the location and distribution of the target proteins CSF1 and CSF1R in the testis and cryptorchidism, and the results show that CSF1 and CSF1R have strong positive expression in SCs, and the positive expressions of CSF1 and CSF1R in cryptorchidism were stronger than those in the testis (Figure 5B–E).

### 3.6. Verification of Sertoli Cell Purity and Validation of Predicted Targeting of CSF1 Gene by Novel-m0230-3p

**Figure 6:**
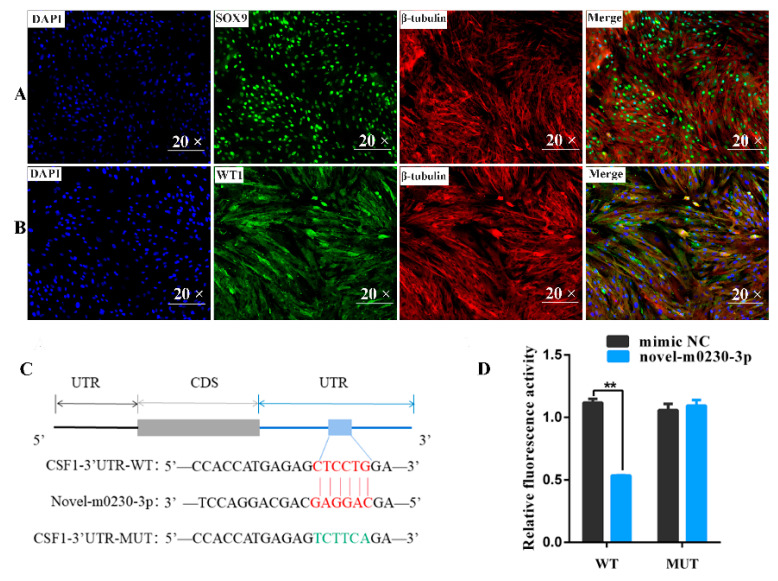
Targeting relationship between target gene CSF1 and novel-m0230-3p. (**A**) IF staining identified isolated yak SCs using antibodies against SOX9 (green) and β-tubulin (red); magnification, 20×. (**B**) Immunofluorescence staining identified isolated yak SCs using antibodies against WT1 (green) and β-tubulin (red); magnification, 20×. (**C**) Binding site of CSF1 and novel-m0230-3p. (**D**) Luciferase activity in SCs after co-transfection with mimics of novel-m0230-3p (100 nM) or mimic NC (100 nM) and pmirGLO-CSF1 3′-UTR-WT (400 ng) or pmirGLO-CSF1 3′-UTR-MUT (400 ng). Values represent mean ± SD; *n* = 3, ** *p* < 0.01.

Both SOX9 and WT1 are marker proteins for Sertoli cells. SOX9 is expressed in the nucleus, while WT1 is expressed in the cytoplasm. Here, we used these two key proteins to identify the purity of primary yaks’ SCs, and β-tubulin was used to stain the cytoskeleton. From the IF results, the nuclei are stained blue and the β-tubulin is stained red (Figure 6A,B). The WT1 AND SOX9 are stained green separately. The results of IF show that almost all cells in the field of view could be labeled with green fluorescence whether SOX9 was used as a nuclear protein or WT1 as a cytoplasmic protein, which show the high purity of the separation and purification of primary SCs, which can be used in subsequent cell transfection (Figure 6A,B). Binding seed sequences in alignments of the CSF1 3′-UTR and novel-m0230-3p were predicted using TargetScan (Version 7.2) and miRanda (V0.10.73) bioinformatics software. The CSF1-MUT group showed six mutated nucleotides in the CSF1 3′-UTR (Figure 6C). The activity of the luciferase reporter in the CSF1 3′-UTR-WT + novel-m0230-3p mimic group was significantly lower than that in the CSF1 3′-UTR-WT + mimic NC group (*p* < 0.01). In contrast, there was no significant difference between the mimic NC and novel-m0230-3p mimic groups in the CSF1 3′-UTR-MUT group. These data confirm that CSF1 is specifically targeted and inhibited by novel-m0230-3p in yak SCs (Figure 6D). 

### 3.7. Transfection Efficiency of Novel-m0230-3p and Expression of CSF1 in Yak Sertoli Cells

**Figure 7:**
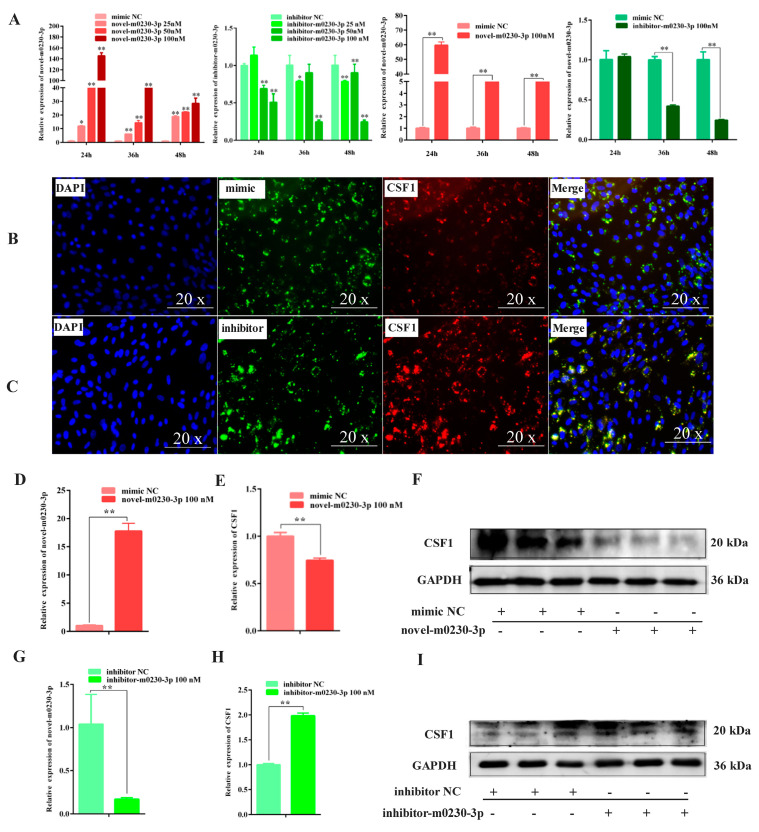
Regulation of CSF1 by novel-m0320-3p. (**A**) Optimal transfection efficiency of mimics and inhibitors of novel-m0230-3p was explored at different concentrations and transfection times. Values represent mean ± SD; *n* = 3. * p < 0.05, ** *p* < 0.01. (**B**) Localization of CSF1 protein and novel-m0230-3p mimic in SCs was analyzed by immunofluorescence staining. CSF1 was colored red, mimic is shown in green, and nuclei were counterstained with DAPI (blue); magnification, 20×. (**C**) Localization of CSF1 protein and novel-m0230-3p inhibitor in SCs was analyzed by immunofluorescence staining. CSF1 was colored red, inhibitor is shown in green, and nuclei were counterstained with DAPI (blue); magnification, 20×. (**D**) mRNA expression of novel-m0230-3p after transfection of 100 nM mimic into SCs for 48 h. Values represent mean ± SD; *n* = 3. ** *p <* 0.01. (**E**,**F**) mRNA and protein expression of CSF1 after transfection of 100 nM mimic into SCs for 48 h. ** *p* < 0.01. (**G**) mRNA expression of novel-m0230-3p after transfection of 100 nM inhibitor into Sertoli cells for 48 h. Values represent mean ± SD; *n* = 3. ** *p* < 0.01. (**H**,**I**) mRNA and protein expression of CSF1 after transfection of 100 nM inhibitor into SCs for 48 h. Values represent mean ± SD; *n* = 3. ** *p* < 0.01.

The optimal point of transfection time and concentrations for novel-m0230-3p and inhibitor-m0230-3p were determined before formal experiments. From the qRT-PCR analysis performed before the experiment, the expression levels of novel-m0230-3p were found to be increased in the SCs to varying degrees at various times, while the expression levels of inhibitor-m0230-3p were decreased. A comprehensive assessment showed that both novel-m0230-3p and inhibitor-m0230-3p had their highest transfection efficiencies at 48 h. Ultimately, 48 h and 100 nM were chosen as optimal conditions for novel-m0230-3p and inhibitor-m0230-3p (Figure 7A). 

The IF analysis showed that when yak SCs were transfected with novel-m0230-3p and inhibitor-m0230-3p, the mimic and inhibitor had green fluorescence, while the nuclei were stained blue and CSF1 was stained red (Figure 7B,C). From the comparison of the IF results, the upregulation of novel-m0230-3p expression after transfection mimic and reduced the fluorescence intensity of CSF1 (Figure 7B), while the transfection of the inhibitor-m0230-3p increased its fluorescence intensity (Figure 7C). In addition, the qRT-PCR and Western blotting results showed an increased expression of novel-m0230-3p and decreased expression of CSF1 in the group transfected with novel-m0230-3p compared to the cells transfected with the mimic NC at the same concentration (Figure 7D–F). In contrast, the expression of novel-m0230-3p was decreased, while the expression of CSF1 was increased following transfection with inhibitor-m0230-3p (Figure 7G–I). These results demonstrate the successful transfection of both the novel-m0230-3p and the inhibitor-m0230-3p into yak SCs, and that the fluorescence intensity and expression levels of CSF1 were attenuated by the targeted binding of novel-m0230-3p. 

### 3.8. Novel-m0230-3p Regulated Adhesion and Tight Junctions in Sertoli Cells via CSF1/CSF1R/Ras Pathway

**Figure 8:**
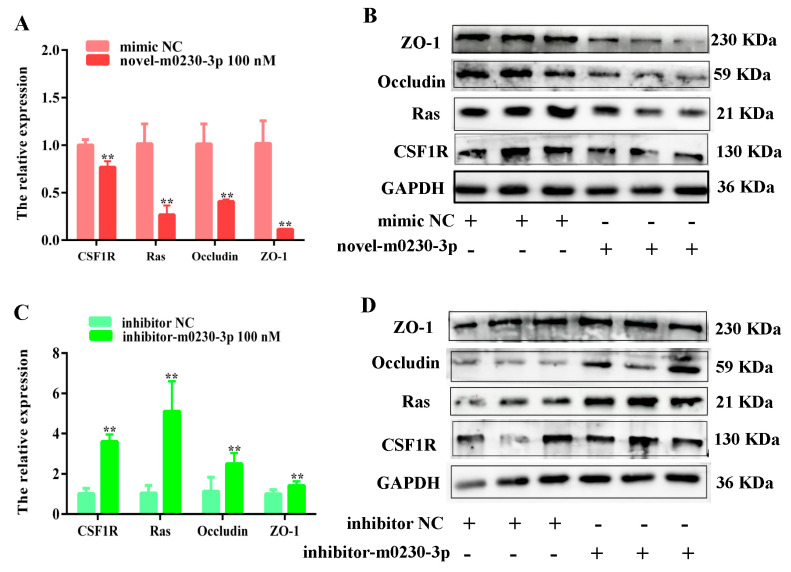
Novel-m0230-3p regulates CSF1 expression via CSF1/CSF1R/Ras signaling pathway to affect cellular TJs and adhesion. (**A**) mRNA expression of CSF1R, Ras, Occludin, and ZO-1 was measured by qRT-PCR after transfection of 100 nM mimic into SCs for 48 h. Values represent mean ± SD; *n* = 3. ** *p* < 0.01. (**B**) Protein expression of CSF1R, Ras, Occludin, and ZO-1 was assessed by Western blotting after transfection of 100 nM mimic into SCs for 48 h. (**C**) mRNA expression of CSF1R, Ras, Occludin, and ZO-1 was measured by qRT-PCR after transfection of 100 nM inhibitor into SCs for 48 h. Values represent mean ± SD; *n* = 3, ** *p* < 0.01. (**D**) Protein expression of CSF1R, Ras, Occludin, and ZO-1 was assessed by Western blotting after transfection of 100 nM inhibitor into SCs for 48 h.

We then explored the specific mechanisms by which novel-m0230-3p and CSF1 affected cell adhesion and TJs. It is known that Occludin is an important integral membrane protein localized at TJs and that it is directly related to ZO-1 [29]. Ras plays key roles in regulating cell–cell contacts. The expression of molecules associated with the CSF1/CSF1R/Ras signaling pathway was assessed using qRT-PCR and Western blotting to explore the mechanism underlying the regulation of cell adhesion and TJs via the novel-m0230-3p/CSF1 axis. The qRT-PCR results showed that the mRNA expression levels of Ras, Occludin, and ZO-1 were significantly decreased in the novel-m0230-3p-mimic group compared with those in the mimic NC group, and the mRNA expression level of CSF1R, the CSF1 receptor, was decreased, too (*p* < 0.05, *p* < 0.01) (Figure 8A). The Western blotting results indicate that the protein expression levels of CSF1R, Ras, Occludin, and ZO-1 were significantly decreased in the novel-m0230-3p-mimic group compared with those in the mimic NC group (Figure 8B). By comparison, both mRNA and protein expressions were opposite to those seen in the inhibitor group. Specifically, the mRNA levels of CSF1 were significantly increased, and those of Ras, Occludin, and ZO-1 were remarkably enhanced in the inhibitor-m0230-3p group compared with those in the inhibitor NC group (*p* < 0.05) (Figure 8C). The protein expressions of Ras, Occludin, and ZO-1 were significantly induced, and that of CSF1R was increased (Figure 8D).

## 4. Discussion

Solving reproductive problems and boosting fertility is a pressing concern for the yak, a unique and rare high-altitude species. Cryptorchidism, or undescended testes, is one of the most common genital malformations in males and is known to impair spermatogenesis and the development of testes [35]. In this study, we compared unilateral normal testis and cryptorchidism samples from the yak. Unilateral cryptorchidism appears as a distinct developmental arrest. Compared with the normal testis, the weight of the unilateral cryptorchidism was 100 g lighter. As the key components of the microenvironment of spermatogonial stem cells (SSCs), SCs provide support and nutrition for the growth of germ cells throughout spermatogenesis [36]. Most importantly, SCs form BTB, which protects autoimmunity germ cells from the host immune system. BTB consists of TJs, gap junctions, desmosomes, and ectoplasmic specialization (ES), with TJs representing the major physical component [37]. Previous studies have shown that the damage and disruption of SCs’ morphology and BTB have been observed in cryptorchidism [38,39]. Spermatogenesis is a complex process involving the regulation of many functional genes, which is inseparable from the normal operation of the BTB. Our H&E-stained histological sections revealed the presence of severe damage in cryptorchidism compared with the normal testis, including disrupted TJs between SCs. mRNA and protein measurements showed marked reductions in the expression of β-catenin and ZO-1 in cryptorchidism, which further suggested that the structure and function of the BTB was impaired in cryptorchidism. MiRNAs are involved in a wide range of biological processes, including reproduction, development, differentiation, proliferation, and aging [19], and a study has shown that miRNA expression changes with both the development of the testis itself and spermatogenic cells [40]. For example, miR-471-5p mediates the expression of DSC2, Occludin, and claudin3 to participate in the regulation of adhesion and affect the functional integrity of BTB [41]. The miR-23b targets PTEN and EPS15 and downregulates FAK expression to inhibit cell adhesion and TJs between SCs [42]. 

However, due to the relative rarity of yak, only a few studies have investigated the biological functions and regulatory networks involved in yak reproduction. In this study, using the power of RNA-seq, we analyzed and compared the miRNA expression profiles between normal testis and cryptorchidism in yak. These profiles were further integrated with mRNA expression profiles to construct miRNA-target regulatory networks, from which key miRNAs and their target mRNAs involved in cryptorchidism were selected. An analysis of the roles of these miRNAs in cryptorchidism showed that the DE target genes were mainly involved in biological processes or pathways associated with cell adhesion, tight junctions, the immune response, and metabolism, suggesting that these factors may directly or indirectly affect the occurrence of cryptorchidism. A further analysis of miRNA and mRNA expression levels revealed that CSF1 was a key factor in cell adhesion and TJs. 

**Figure 9:**
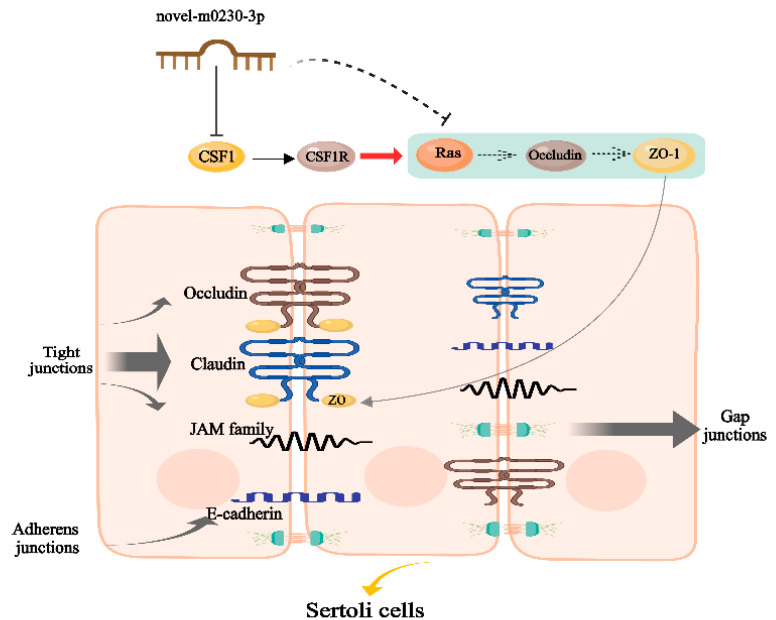
Regulatory effects of novel-m0230-3p on cell TJs and adhesion via CSF1/CSF1R/Ras signaling pathway in yak SCs. Abbreviations: CSF1: colony-stimulating factor 1; CSF1R: colony-stimulating factor 1 receptor; ZO-1: TJP1 (ZO1) tight junction protein 1.

TJs are associated with Occludin, ZO-1, and ZO-2 [43]. Ras is a signal-transducing factor for various membrane receptors and is thought to influence cell adhesion, TJs, and the cytoskeleton via alterations in downstream signaling pathways [44]. A previous study has shown that ZO-1 is localized in vitro in TJs, and this location is regulated by activated Ras [45]. These findings suggest that Ras can modulate the function of ZO-1 to influence TJs [45,46]. Hence, we want to investigate whether TJs in SCs are regulated by novel-m0230-3p through the CSF1-mediated CSF1/CSF1R/Ras signaling pathway. In this study, we found that the mRNA and protein expression of Ras was decreased after the transfection of SCs with mimics of novel-m0230-3p and were increased after the transfection of inhibitor-m0230-3p. Similarly, the expressions of both Occludin and ZO-1 corresponded with that of Ras. These results show that mimics of novel-m0230-3p inhibited the expression of the target gene CSF1, and CSF1 can promote cell TJs by increasing the expressions of Ras, Occludin, and ZO-1 via the CSF1/CSF1R/Ras signaling pathway (Figure 9). These findings suggest that the novel-m0230-3p targeted CSF1 and mediated the CSF1/CSF1R/Ras signaling pathway to inhibit the expression of ZO-1 and Occludin, and both are key proteins of cells and TJs in SCs, resulting in the occurrence of yak cryptorchidism.

At present, there is very little research related to yaks, and there are still big challenges to further explore their reproductive physiology. For one thing, there are no yak-specific antibodies on the market, which is causing great trouble for our scientific research. For example, the antibodies used in this manuscript are all mouse or rabbit monoclonal antibodies, which have shown shortcomings such as low antibody specificity and weak binding. Furthermore, the species differences are also worth considering. Furthermore, yak cell lines have received less attention, and related experiments on yak cells often rely on the culture and purification of primary cells. Sertoli cells, as the most important somatic cells in the testes, play a central role in spermatogenesis and are indispensable objects in the study of the reproductive physiology of the yak. While we have been successful in purifying yak SCs, these primary SCs grow slowly, are easily differentiated, have a finite generation to proliferation, and are difficult to transfect in vitro compared to mature cell lines. Therefore, in the future, it is urgent and imperative to prepare stable, rapidly growing, mutation-free yak cell lines as an important tool for the study of yak reproductive physiology. 

## 5. Conclusions

In conclusion, this study focuses on the regulation of target genes by miRNAs and their effects on testicular development and reproductive physiology in the yak. We know that the BTB and cell TJs are essential for the development of the testes, and both are destroyed in cryptorchidism. In this study, we present a model of the miRNA-mRNA network mediated by CSF1/CSF1R/Ras signaling to show its regulatory role in cell TJs. First, the expression of CSF1 is negatively regulated by novel-m0230-3p. Then, CSF1 regulates related molecules such as CSF1R and Ras in the CSF1/CSF1R/Ras signaling pathway, ultimately influencing changes in Occludin and ZO-1, which are key proteins in the BTB and associated with healthy testicular development. We hope that this study will provide an academic basis for elucidating the molecular regulatory mechanisms underlying testicular development in the yak, as well as theoretical evidence for further investigations on the molecular markers of male yak fertility.

## Data Availability

The data are available in the Sequence Reads Archive (SRA): https://www.ncbi.nlm.nih.gov/sra/PRJNA1076167 (accessed on 8 January 2024).

## Supplementary Materials

The following supporting information can be downloaded at: https://www.mdpi.com/article/10.3390/cells13151304/s1, Table S1: Information on primer sequences for PCR; Table S2: 575 different expression mRNA; Table S3: 103 different expressionTestis-vs-Crypto. Table S4: miRNA-mRNA.
