# Supplementary material for: The Novel-m0230-3p miRNA Modulates the CSF1/CSF1R/Ras Pathway to Regulate the Cell Tight Junctions and Blood–Testis Barrier in Yak
Source: Cells. 2024 Aug 5;13(15):1304. doi: 10.3390/cells13151304 (PMC11311379; doi:10.3390/cells13151304)
Supplement: Supplementary file 1 [file cells-13-01304-s001.zip › Table S1 Information on primer sequences for PCR.pdf]

Table S1. Information on primer sequences for PCR

Name	Gene sequence (5′–3′)	Tm (℃)
ZO-1 (TJP1)	F: GAGGACAGTTACGACGAGG<br>R: CAGGGTGACTTTGGTGGG	59
Occludin	F: GAACGAGAAGCGACCGTATCC<br>R: CTTGCTCTGCCCGCCTTG	59.5
Ras	F: TGTGGTAGTTGGAGCTGGTG<br>R: TCATGTACTGGTCCCTCATTG	55
Rassf6	F: CGCATTAGTGACTTGGACAGG<br>R: GGAGATTCAGGCTCTTCGTTT	57
CSF1	F: GGAGGTGTCGGAGAACTGT<br>R: TCTTTGAAGCGCATGGTAT	54
CSF1R	F: TGAACAAGACCTGGACAA<br>R: GAGCCCGAGTGAGAAGA	52.5
β-catenin	F: CAGGGAAGACATCACTGAGCCT<br>R: GCACGAACCAGCAACTGAACTA	59
IGPB5	F: GCTGAGAACACTGCCCATCC<br>R: CCTTTGCGGTCACAGTTGG	58
IRF1	F: AATACAGCCCCGATACCTTC<br>R: TTCCCTTCCTCGTCCTCAT	55.3
ITGB7	F: CCAGAGGGTAAAGGGAGCA<br>R: CCGATAAGCGAGGACCAA	56.5
LCP2	F: CAAGTCCAGCGGTTTCC<br>R: GGGTGGTGGCTCGTAAT	54.5
GAPDH	F: GCTGGTGCTGAGTATGTGGTG<br>R: GCTGACAATCTTGAGGGTGTTG	58
TNSSF10	F: CGCATTAGTGACTTGGACAGG<br>R: GGAGATTCAGGCTCTTCGTTT	55
ADD2	F: TGAAGAGGCTAAACTGACGG<br>R: GAAGGATGGGGTACGGAAT	55
SYK	F: ACCTCATCCGAGAATACG<br>R: CTGTGGTGGCTATCAGTTT	53
CD27	F: GGGAGGATGGTGTTGTCAGA<br>R: TTCAGCATAAGGTAAGTGGGAG	56
PTPRC	F: ACATCGCAGCACAAGGTCC<br>R: TGAGCAGCAGGTGAGGGTC	58
HSPB8	F: GGTAAAGACCAAGGACGGAT<br>R: GGAAAGGGAGGCAAACACT	55.3

INSRR	F: CCACCAGGGTAGTTGTCT<br>R: ACGGAGGTTGAGGATGA	53.5
LGR5	F: TTTTCCACCTTGCCATCT<br>R: GTGCTGTTGTCGCCTTTA	52
TINAG	F: TCCCAGCATGTATGCCTTGT<br>R: CGGTATTTGAAACCCTCCTCTA	55.5
SOX10	F: CCAAAGCCCAGGTGAAGA<br>R: GGCAGACTGAGGGAGGTGT	57.5
TLR4	F: CTGCCTTCACTACAGGGACTT<br>R: TGGGACACCACGACAATAAC	56.5
LPXN	F: CCCTTCCGTTCCTGATGACA<br>R: TGCGGCTGCTGAGGTTTTA	56
miR-485-y	F: GCGAATACCTCGGACCCT	58
miR-670-z	F: ACCGTCGTATCCAGTGCG	58
miR-1599-z	F: GCGAATACCTCGGACCCT	60
miR-7890-y	F: TTCTGGGTGCTGAAAACGTC	60
novel-m0108-3p	F: CGTGGGAAGACATGGAGATAG	57.8
novel-m0230-3p	F: AGCGTGAGCAGGAGCAGC	60
U6	F: CGAGGATGTGAAGACACCAAGAC	59
mimic NC	F: UUGUACUACACAAAAGUACUG<br>R: GUACUUUUGUGUAGUACAAUU	58
novel-m0230-3p	F: AGCAGGAGCAGCAGGACCU<br>R: GUCCUGCUGCUCCUGCUUU	58
