# Supplementary material for: The Novel-m0230-3p miRNA Modulates the CSF1/CSF1R/Ras Pathway to Regulate the Cell Tight Junctions and Blood–Testis Barrier in Yak
Source: Cells. 2024 Aug 5;13(15):1304. doi: 10.3390/cells13151304 (PMC11311379; doi:10.3390/cells13151304)
Supplement: Supplementary file 1 [file cells-13-01304-s001.zip › 伦理审查表 牦牛.pdf]

甘肃农业大学动物实验伦理审查申请书

Examine application for animal experiment ethics executed in Gansu Agricultural University

申请日期(applicant date)：2023 年(year) 6 月(month) 1 日(day)

申请编号(applicant number)：GSAU-Eth-VMC-2023-036

<table>
<tr><td colspan="3">研究用途(Research purpose)：牦牛睾丸支持细胞分离纯化、生殖生理等研究</td></tr>
<tr><td colspan="3">申请人姓名(applicant name) 王琪 技术职称(Technical title)：讲师 岗位证书编号(Post certificate number)：动字（）第 号</td></tr>
<tr><td colspan="3">项目负责人(Project Leader)：王琪 技术职称(Technical title)：讲师 联系电话(Contact phone)：0931-7631236
手机(Mobile phone)：15294157985</td></tr>
<tr><td colspan="3">申请者单位或部门(Applicant unit or department)：动物医学院 负责人(Leader)：成述儒</td></tr>
<tr><td rowspan="4">拟进动物情况</td><td colspan="2">动物来源(Animal resources)：各合作牦牛养殖场、实验动物研究机构等</td></tr>
<tr><td colspan="2">品种品系(Species or strains)：牛科牛属 等级(Grade)：依具体试验而定 规格(Specifications)：幼龄及成年</td></tr>
<tr><td>数量(quantity)：依具体试验而定
只(其中：♀ 只；♂ 只) 依具体试验而定</td><td>申购日期(Date of purchase)：依具体试验而定
年(year) 月(month) 日(day)</td></tr>
<tr><td>进驻日期(Entering date)：依具体试验而定
年(year) 月(month) 日(day)</td><td>结束日期(End date)：依具体试验而定
年(year) 月(month) 日(day)</td></tr>
</table>

遵守实验动物福利原则的声明：本人已阅读了《甘肃农业大学实验动物伦理委员会章程》，本人及其项目组成员将严格遵守国际通行的动物福利和伦理准则，贯彻执行国家和甘肃省有关实验动物管理法律、法规和政策，善待实验动物。

Declaration of compliance with the principles of animal welfare: I have read the "constitution of the experimental animal ethics committee of Gansu Agricultural University", I and the project team members will strictly abide by the international animal welfare and ethical standards, carry out the relevant laws, regulations and policies on the management of laboratory animals in Gansu Province, and care laboratory animals.

项目负责人签名(Project Leader signature)：王琪

实验要点,包括：实验目的、实验方法、观测指标、实验结束后处置动物的方法等(Experimental points, including: the experimental purpose, experimental methods, observation indicators, assays for animal sacrifice, etc. after the end of the experiment)

实验目的(Experimental purpose)：用于牦牛睾丸支持细胞分离及纯化。

实验方法(Experimental methods)：各种生物学方法。

观测指标(Observation indicators)：依具体试验而定。

实验结束后处置动物的方法（包括处置的方式等）(Assays for animal sacrifice, etc. after the end of the experiment, including methods to end animal life, etc.)：按国际动物福利法和伦理法处置。

幼龄动物采取手术，取下睾丸后留置养殖场继续养殖；成年动物尽量在屠宰前进行相关手术，术后留给养殖场进行后续处理。

手术过程中动物福利保障措施（包括减轻动物痛苦措施等）(Safeguard measures for animal welfare during operation, including measures to alleviate the pain of animals, etc.)：按国际动物福利法执行。

手术局部麻醉，简洁、迅速、准确地完成实验，尽力保证动物术中、术后及运输过程中免受痛苦。

危险物品、药品安全处置措施（包括针头、有害药品的安全处置措施等）(Safety management measures of hazardous substances, drug, including safety management measures of needles, the safe disposal of hazardous drugs, etc.)：由专业生物医药及危化品处理公司回收处置。

集中放置于医疗废物规定暂存处，交由专业医疗废物处置机构进行无害化处理。

审查依据

1. 该项目是否必须用实验动物进行实验，即能否用计算机模拟、细胞培养等非生命方法替代动物或用低等动物替代高等动物进行实验。(1.Whether the project must be carried out with laboratory animals, Whether higher animal used to animal experiments can be substituted by computer simulation, cell culture, other nonlife alternatives or low animal) 必须使用实验动物，不能用计算机模拟、细胞培养等非生命方法替代动物或用低等动物替代高等动物进行实验。
2. 表中所填申请人资格和所用动物的品种品系、质量等级、规格是否合适，能否通过改良设计方案或用高质量的动物来减少所用动物的数量。(2. whether the applicant qualifications, animal strains or lines used, quality grades, specifications are suitable, whether the number of animals used can be reduced by improving the design scheme or the use of high quality animals) 合适，不能通过改良设计方案或用高质量的动物来减少所用动物的数量。
3. 能否通过改进实验方法、调整实验观测指标、改良处置动物的方法，来优化实验方案、善待动物。(3.Whether animal experiment can be optimized and animal can be cared through improving experimental method, adjusting the observation index, and improving the disposition of an animal)不能通过改进实验方法、调整实验观测指标、改良处置动物的方法来优化实验方案、善待动物，但已做了最大化的努力。
4. 保证动物福利的相关措施是否落实。(4. Whether the relevant measures to ensure implementation of animal welfare were carried out) 已落实。

主管单位负责人意见(Comments of the person in charge of the competent authority)：

审查通过。

签章(signature) [illegible]

动物医学院

2023年(year) 6月(month) 1 日(day)

伦理委员会评审决议(Ethics committee review decision)： 审查通过。

伦理委员会主任委员签章(Ethics committee chairman signature) 張勇

2023年(year) 6月(month) 1 日(day)

说明： 1.此表一式二份，正反面打印。在伦理审查通过后返还项目负责人一份，伦理委员会留存一份。2.编号由伦理委员会秘书填写。3.联系电话填本项目申报联系人的电话。4.实验要点只写摘要。5.申请单位或部门填到科室，主管单位签章为学院主管科研的领导签章。

Note: 1. this form has two copies, positive and negative print.one will be returned to the project leader, another will be retained in the ethics committee after the ethical review is passed by the committee of ethics. 2. The applicant number will be given by the ethics committee secretary. 3 Contact phones should be filled by the project applicant. 4.The item of “Experimental point” should be filled only with project summary. 5. The item of “application units or departments” should be filled with department; the signature of the competent unit should be performed by the leader in charge of scientific research in the college or institute.
